# Inhibiting of GRASP65 Phosphorylation by DL-3-N-Butylphthalide Protects against Cerebral Ischemia-Reperfusion Injury via ERK Signaling

**DOI:** 10.1155/2018/5701719

**Published:** 2018-08-01

**Authors:** Bei-lei Zhu, Chen-long Xie, Ning-ning Hu, Xin-bo Zhu, Chun-feng Liu

**Affiliations:** ^1^Department of Neurology and Suzhou Clinical Research Center of Neurological Disease, The Second Affiliated Hospital of Soochow University, Suzhou 215004, China; ^2^Department of Neurology, The First Affiliated Hospital of Wenzhou Medical University, Wenzhou 325000, China; ^3^Department of Medicine, First Clinical Medicine School, Wenzhou Medical University, Wenzhou 325035, China; ^4^Department of Pharmacy, Pharmacy School, Wenzhou Medical University, Wenzhou 325035, China

## Abstract

**Background and Purpose:**

The aim of this study was to explore the role of DL-3-n-butylphthalide (NBP) in cerebral ischemia-reperfusion injury (CIRI) mice model. The involvement of extracellular signal-regulated kinase (ERK) signaling pathway was also investigated.

**Methods:**

All mice were divided into five groups: sham-operated group, CIRI group, NBP pretreatment group, NBP treatment group, and NBP pretreatment + treatment group. The CIRI mice model was established by the use of the Pulsinelli four-vessel occlusion method. Pretreatment mice received NBP (90 mg/kg/d) three times a day within four days before reperfusion by gavage. Treatment mice received NBP (90 mg/kg/d) three times a day within five days after reperfusion by gavage. We detected the infarction area, the neurological severity, and the superoxide dismutase and malondialdehyde levels. Furthermore, we observed the expressions of GRASP65, phosphorylation of GRASP65 (pGRASP65), ERK, and phosphorylation of ERK (pERK) by the use of Western blotting.

**Results:**

The result showed that the ERK pathway was activated in response to CIRI. NBP decreases the expressions of pERK and pGRASP65 following CIRI. Additionally, NBP could decrease MDA and increase SOD level in brain tissues. Decreased infarct volume was also observed in the NBP group. Thereby, NBP inhibited the activation of the ERK pathway induced by CIRI and reduced the GRASP65 phosphorylation.

**Conclusions:**

The current finding suggested that NBP protected the cerebrum from CIRI mediated by inhibiting the ERK signaling pathway and subsequently reducing GRASP65 phosphorylation.

## 1. Introduction

Cerebral ischemia-reperfusion injury (CIRI), characterized by aggravated nerve damage and dysfunction, is the most common complication to occur in stroke patients following thrombolytic therapy. CIRI accounts for 85% of all stroke patients leading to substantial health-care burden in patients and society [1]. To date, the exact mechanism of CIRI remains unclear. Therefore, full understanding of potential mechanism of CIRI and identification of effective treatment strategy for CIRI are of great clinical significance.

Previous studies have demonstrated that oxidative stress plays a key role in the pathophysiology of CIRI by the overproduction of free oxygen radicals, resulting in substantial cell apoptosis and death [2, 3]. In the process of oxidative stress, the Golgi apparatus (GA) is a key organelle that participates in signal transduction, homeostasis, and cell apoptosis; this effect is named as the Golgi apparatus stress (GAS) [4, 5]. Extracellular signal-regulated kinase (ERK) belongs to the system of mitogen-activated protein kinase (MAPK). A growing number of evidence has revealed that ERK pathway is involved in the oxidative stress, participating in the neuronal damage caused by oxidative stress [6]. Furthermore, previous research reported that the phosphorylation of the Golgi reassembly and stacking protein 65 (GRASP65) was mediated by ERK, resulting in cisternae coming apart [7].

**Figure 1:**
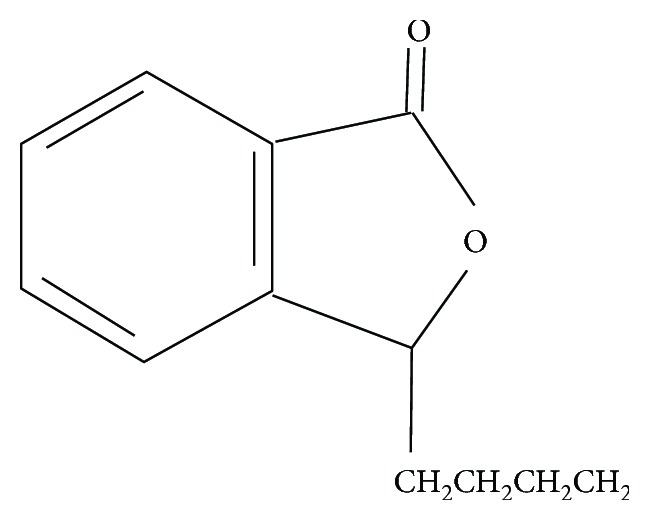
Chemical structures of DL-3-n-butylphthalide.

DL-3-n-butylphthalide (NBP) is a racemic compound developed from L-3-n-butylphthalide, which was initially derived from seeds of *Apium graveolens* (Figure 1). Studies had reported that NBP can protect against ischemic cerebral injury by attenuating oxidative stress damage [8], suppression of inflammatory reactions, and neuronal apoptosis [9]. In addition, clinical trials have demonstrated the efficacy of NBP in the treatment of acute ischemic stroke patients [10, 11]. As mentioned earlier, the GA was involved in oxidative stress. However, whether NBP can exert a protective effect on the GA in CIRI remains unknown.

Our purpose of the current study was to explore the role of NBP in CIRI mice. In addition, the mechanisms of NBP attenuated the GAS induced CIRI via the ERK signaling pathway were also investigated.

## 2. Materials and Methods

### 2.1. Chemicals

The NBP soft capsules were purchased from the CSPC Pharmaceutical Group Company Co. Ltd. (Hebei, China). NBP capsules (0.1 g) were diluted with soybean oil into 2.5 mg/mL preparations and were stored at 4°C. Chloral hydrate was purchased from the Sinopharm Chemical Reagent Co., Ltd. (Shanghai, China). The assay kits of superoxide dismutase (SOD), malondialdehyde (MDA), total protein, and bicinchoninic acid (BCA) protein were purchased from the Nanjing Jiancheng Bioengineering Institute (Nanjing, China). Anti-GRASP65 (sc-398363) and antiphospho (p)-GRASP65 (sc-389542) antibodies were purchased from the Santa Cruz Biotechnology, Inc. (Dallas, TX, USA). Anti-ERK (4696S) and anti-pERK (9106L) antibodies were purchased from the Cell Signaling Technology, Inc. (Danvers, MA, USA).

### 2.2. Experimental Animals

Fifty males and fifty females, specifically pathogen-free, ICR mice (23–27 g) were provided by the experimental animal center of Wenzhou Medical University (Wenzhou, China). Mice were housed in a specific pathogen-free environment and were allowed free access to food and water intake. The houses of mice were under controlled temperature (21–23°C) and relative humidity (51–61%) with a light/dark cycle of 12 h. All mice were randomly divided into 5 groups (10 male and 10 female each group) as follows: sham-operated (group A); control (group B); NBP (group C), including pretreatment (C1), treatment (C2), and pretreatment + treatment (C3). This study protocol was approved by the experimental Animal Ethics Committee of Wenzhou Medical University.

### 2.3. Modeling Method

The Pulsinelli four-vessel occlusion method was used to establish the mice model of cerebral ischemia [12]. Mice were anesthetized using 1.5% pentobarbital (0.1 mg/20 kg) by intraperitoneal injection. We made a 1.5 cm incision in the middle of the neck and carefully separated the jugular muscles. Following that, we occluded the bilateral common vertebral arteries using electrocoagulation and sutured the incision. Then, the mice were allowed to recover.

On the second day, we repeated the aforementioned steps and occluded the common carotid arteries for 5 minutes using small bulldog clamps. Group A underwent the surgery without occlusion. Group A and group B received soybean oil (90 mg/kg/d) by gavage. Group C1 and group C3 were pretreated with gavage administration of the NBP at 90 mg/kg/d three times a day within four days before reperfusion. Group C2 and group C3 were treated with gavage administration of the NBP at 90 mg/kg/d three times a day within five days after reperfusion.

### 2.4. Neuroethological Assessment

The neurological severity score (NSS) was used to evaluate the neurological damage of mice after 5 days of reperfusion [13].

### 2.5. Specimen Collection and Determination of Infarction Area

After 5 days of establishing the model, mice from each group underwent decapitation. The brain was dissected after craniotomy and perfused with normal saline. The brain was fixed in a 4% paraformaldehyde solution, then was embedded with paraffin. The specimens were sectioned into 5 *μ*m thick slices and stained with hematoxylin and eosin (HE) [14]. During TTC staining, the brain was perfused with normal saline at 4°C for 10 min. The specimens were sectioned with a thickness of 2 mm. Next, we incubated the slices in 2% solution of TTC for half hour under constant temperature (37°C) in a water bath. The areas of cerebral infarction were defined as the unstained areas of the brain and calculated using an image analysis software (Image-Pro Plus, version 6.0; Media Cybernetics, Inc., Rockville, MD, USA) [15].

### 2.6. Measurement of Cerebral SOD and MDA

The brain was homogenized in cold normal saline at 4°C, and the samples were centrifuged at 1006 ×g for 10 min. Supernatants were collected via the MDA and SOD kits. Levels of MAD and SOD were calculated using the Multiskan Spectrum microplate reader according to the readings of optical density at 552 and 550 nm (Thermo Fisher Scientifc, Inc., Waltham, MA, USA).

### 2.7. Western Blot Analysis

The protein concentrations of ERK, pERK, GRASP65, and pGRASP65 in the homogenate samples were measured using Western blotting. Total protein concentrations in the homogenate samples were measured by the BCA protein assay kit. An equivalent amount of protein (20 *μ*g sample/lane) was separated on a 10% SDS-PAGE gel and transferred to a polyvinylidene difluoride membrane. The proteins were detected with antibodies against ERK (1 : 1000), pERK (1 : 1000), GRASP65 (1 : 200), and pGRASP65 (1 : 500). *β*-Actin was used as internal control. Then, the membranes were incubated with secondary HRP-conjugated anti-rabbit (KS001) or anti-goat (KS003) antibodies (dilution, 1 : 3000) for 2 h. The proteins were visualized by the Western blotting chemiluminescence reagent. Blots were quantified using the BandScan software (version 5.0; Glyko, Inc., Novato, CA, USA).

### 2.8. Statistical Analysis

Statistical analyses were performed in the SPSS software 21.0. Data are demonstrated as mean standard deviation (SD). The comparison between different groups was made using ANOVA, followed by the post hoc Tukey test and Bonferroni correction. Neurological severity score was analyzed using the Kruskal-Wallis test. The Nemenyi test was used for multiple comparisons. *P* < 0.05 (2-tailed) was considered as statistically significant.

## 3. Results

### 3.1. Neuroethological Assessment following CIRI

**Figure 2:**
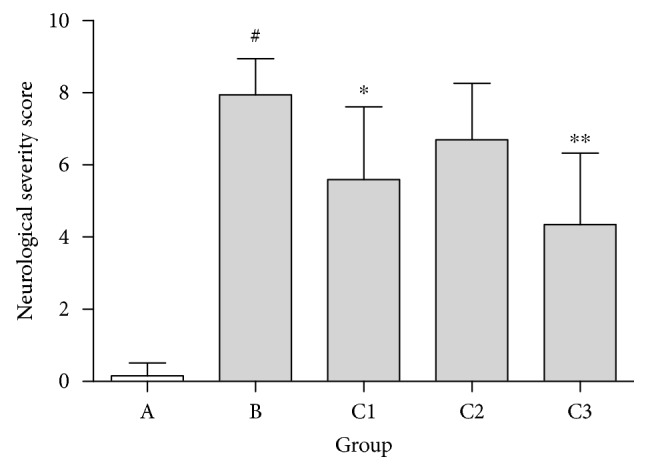
Neurological severity score of each group following CIRI for 5 days. ^#^*P* < 0.01 versus group A; ^∗^*P* < 0.05, ^∗∗^*P* < 0.01 versus group B. Group A: sham-operated; group B: control; group C1: pretreatment; group C2: treatment; group C3: pretreatment + treatment.

Groups C1 and C3 had significantly decreased neurological severity scores than group B (*P* < 0.01; Figure 2). Additionally, groups C1 (*P* < 0.05; Figure 2) and C3 (*P* < 0.01; Figure 2) had lower neurological severity scores than group B.

### 3.2. Effect of NBP on Neurocyte Morphology Changes

**Figure 3:**
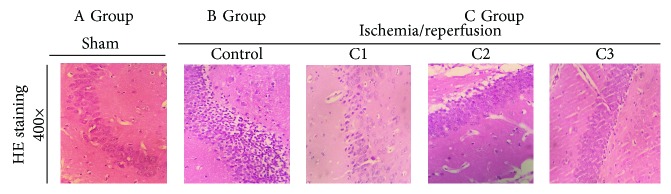
Alteration in the neurocyte morphology of CIRI mice. Stained with HE. Magnification: ×400; HE: hematoxylin and eosin; group A: sham-operated; group B: control; group C1: pretreatment; group C2: treatment; group C3: pretreatment + treatment.

Cells from group A were closely arranged and had normal nuclear morphology, uniformly stained by HE (Figure 3). Conversely, cells from group B were loosely arranged with several abnormal shapes, such as nuclear condensation, interstitial edema, and neuronal degeneration (Figure 3). In mice from the NBP treatment group (group C2), some cells still exhibited nuclear condensation and other abnormal pathological changes. Cells from group C3 had little difference compared with group A. The cellular effects of cells from group C1 were between those observed in groups C2 and C3 (Figure 3).

### 3.3. Effect of NBP on Infarct Area following CIRI

**Figure 4:**
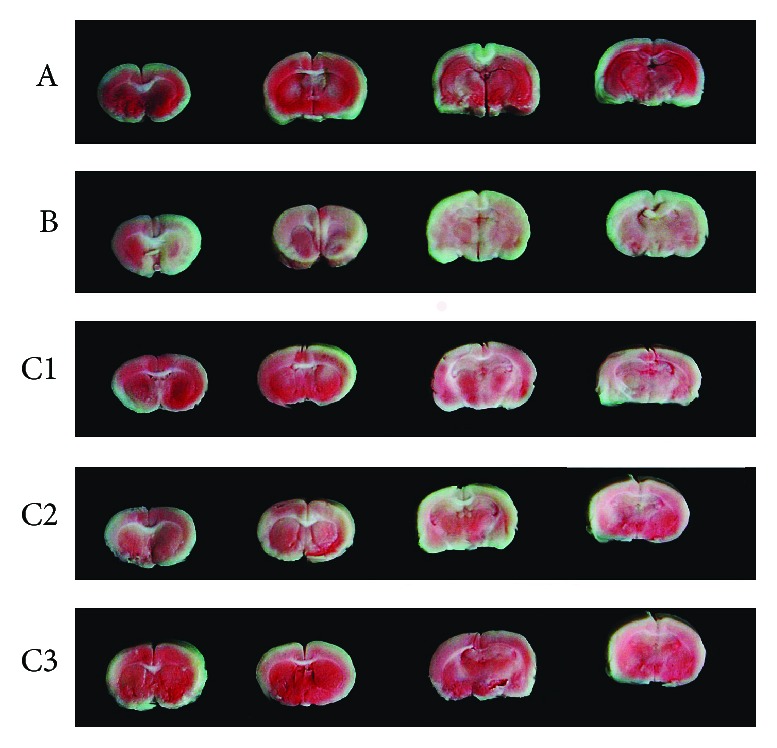
Infarct areas of the brain tissue within 5 days after reperfusion, staining by triphenyltetrazolium chloride. Group A: sham-operated; group B: control; group C1: pretreatment; group C2: treatment; group C3: pretreatment + treatment.

**Figure 5:**
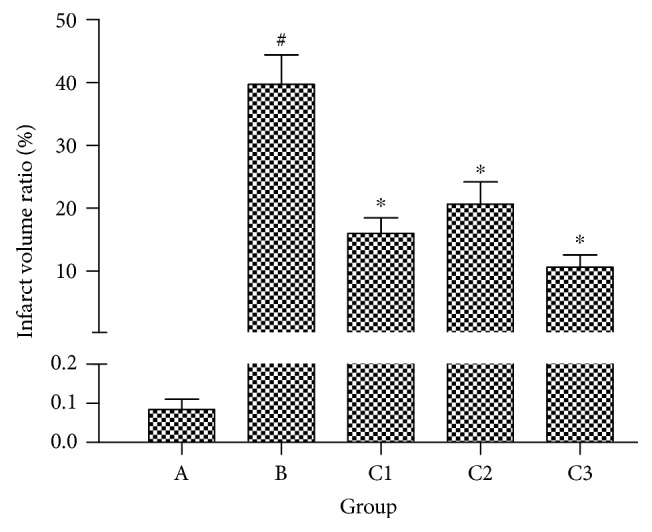
Infarct volume ratio of the brain tissue in each group within 5 days after CIRI. ^#^*P* < 0.01 versus group A, ^∗^*P* < 0.01 versus group B. Data are presented as the mean ± standard deviation; *n* = 7. Group A: sham-operated; group B: control; group C1: pretreatment; group C2: treatment; group C3: pretreatment + treatment.

In the sham-operated group (group A), the brain tissue was uniformly stained by TTC and exhibited no significant infarction. By contrast, group B had significantly increased infarct size than group A and group C (*P* < 0.01; Figures 4 and 5). Furthermore, group C3 had significantly lower infarct size than groups C1 and C2 (*P* < 0.01; Figures 4 and 5). Group C2 also had lower infarct size compared with group C1 (*P* < 0.01; Figures 4 and 5).

### 3.4. Effect of NBP on MDA and SOD

**Figure 6:**
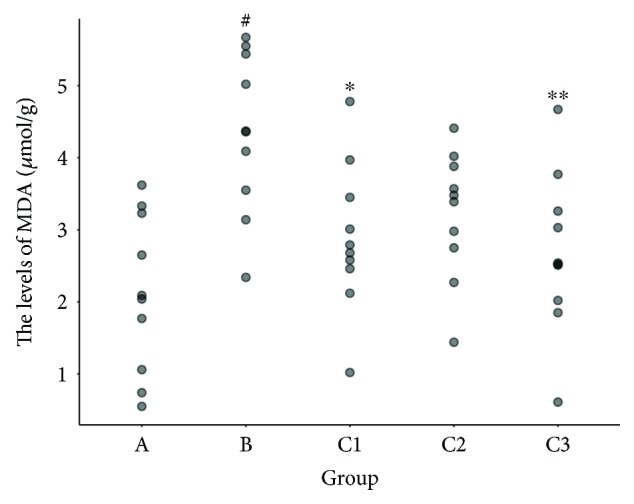
Changes in the levels of MDA in mice cerebral tissue homogenates. ^#^*P* < 0.01 versus group A; ^∗^*P* < 0.05, ^∗∗^*P* < 0.01 versus group B. Data are presented as the mean ± standard deviation; *n* = 10. MDA: malondialdehyde; SOD: superoxide dismutase; group A: sham-operated; group B: control; group C1: pretreatment; group C2: treatment; group C3: pretreatment + treatment.

**Figure 7:**
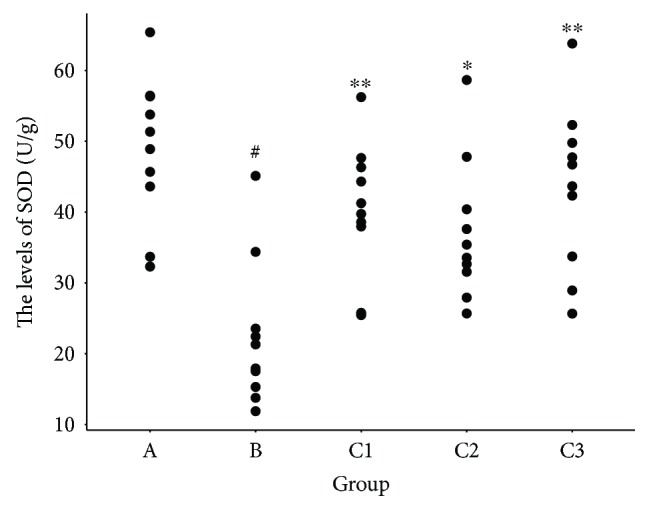
Changes in the levels of SOD in mice cerebral tissue homogenates. ^#^*P* < 0.01 versus group A; ^∗^*P* < 0.05, ^∗∗^*P* < 0.01 versus group B. Data are presented as the mean ± standard deviation; *n* = 10. MDA: malondialdehyde; SOD: superoxide dismutase; group A: sham-operated; group B: control; group C1: pretreatment; group C2: treatment; group C3: pretreatment + treatment.

Mice in the control group had significantly higher MDA levels and lower SOD levels than those in group A (*P* < 0.01; Figures 6 and 7). In addition, group C (groups C1, C2, and C3) had higher SOD levels than group B (*P* < 0.05; Figures 6 and 7). Group C1 and group C3 also had lower MDA levels compared with group B (*P* < 0.05; Figures 6 and 7).

### 3.5. ERK, pERK, GRASP65, and pGRASP65 Levels in Five Groups

**Figure 8:**
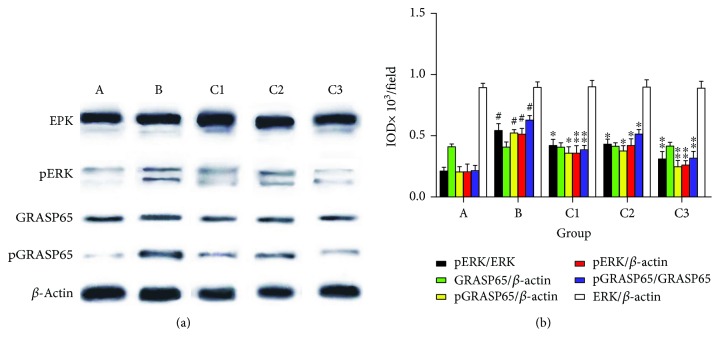
ERK, pERK, GRASP65, and pGRASP65 expressions in mice from each group within 5 days after CIRI. ^#^*P* < 0.01 versus group A; ^∗^*P* < 0.05 versus group B, ^∗∗^*P* < 0.01 versus group B. Data are presented as the mean ± standard deviation; *n* = 10. Group A: sham-operated; group B: control; group C1: pretreatment; group C2: treatment; group C3: pretreatment + treatment; ERK: extracellular signal-regulated kinase; p: phosphorylated; GRASP65: Golgi reassembly and stacking protein 65.

No significant difference was observed in the level of ERK between groups (*P* > 0.05; Figure 8). Nevertheless, the pERK level and the ratio of pERK/ERK in group B were significantly higher than those in group A (*P* < 0.01; Figure 8). Compared with group B, lower ratio of pERK/ERK and lower level of pERK were found in groups C1, C2, and C3 (all *P* < 0.01). This indicated that the activation of the ERK signaling pathway in CIRI was suppressed by NBP.

Similarly, no significant difference was observed in the level of total GRASP65 between groups (*P* > 0.05; Figure 8). Nevertheless, the pGRASP65 level and the ratio of pGRASP65/GRASP65 in group B were significantly higher than those in group A (*P* < 0.01; Figure 8). The results suggest that CIRI may accelerate the phosphorylation of GRASP65. Decreased level of pGRASP65 and lower ratio of pGRASP65/GRASP65 were also found in groups C1, C2, and C3 (all *P* < 0.01) when compared with those in group B. Thus, NBP protects against CIRI via downregulated phosphorylation of GRASP65, and this effect may be mediated by the ERK signaling pathway.

## 4. Discussion

Previous studies have demonstrated that the ERK pathway is related to cellular division and differentiation and is also activated by some pathological conditions such as oxidative stress and cerebral ischemia [16, 17]. Lin et al. reported that cerebral ischemia-reperfusion injury induces the phosphorylation of ERK [18]. In the current study, higher levels of pERK and pERK/ERK were observed in cerebral ischemic mice when compared with sham-operated mice, which are concordant with previous study. The results showed that the ERK pathway was activated in response to CIRI.

The ERK activation is involved in exacerbation of ischemic damage. Numerous studies have reported that the suppression of ERK phosphorylation frequently reduces ischemic injury and infarction [19, 20]. Furthermore, the activation of ERK also promotes inflammatory response and oxidative stress [21]. In this study, we also found that the pERK levels in mice administered with NBP were significantly lower than those of control, indicating that NBP may attenuate the activation of the ERK pathway induced by CIRI. This effect provides further evidence supporting the neuroprotective effect of NBP on CIRI.

Our study revealed that CIRI mice had increased expression of pGRASP65 and pGRASP65/GRASP65 than those of sham-operated mice. GRASPs are membrane proteins served in stacking Golgi cisternae consist of GRASP65 and GRASP55. Specifically, GRASP65 has an intricate role in the morphology alterations of the Golgi apparatus [22, 23]. Furthermore, GRASP65 is phosphorylated on serine-277 by ERK and cyclin-dependent kinase 1 (CDK1) in the process of cell division, resulting in the depolymerization of the Golgi apparatus [7, 24]. Compared to control mice, in NBP-treated mice, the phosphorylation of GRASP65 was reduced. The ERK pathway may mediate this effect and thereby may be involved in the neuronal degeneration after CIRI. When oxidative stress occurs, the Golgi apparatus served as a crucial downstream target organelle of endoplasmic reticulum and mitochondria associated with GRASP65 phosphorylation. Thus, the GAS inhibits the synthesis of essential proteins, exacerbating the CIRI.

Reactive oxygen species (ROS) are the free radicals that originate from oxygen, including superoxide radicals, singlet oxygen, lipid peroxide, hydrogen peroxide, and hydroxyl radicals. These ROS can cause cell and tissue damage during the conditions of stress. MDA is a by-product of lipid peroxidation and often used to measure the status of oxidative stress. Antioxidant enzymes are effective at reducing the uncontrolled formation of free radicals, such as SOD. Several studies have reported that SOD can maintain homeostasis and attenuate the damage caused by ROS during CIRI [25, 26]. Because of its short half-lives, SOD is also used as a marker of antioxidant status. It was found that NBP can play antioxidative and neuroprotective roles in reducing the injury of cerebral ischemia reperfusion [27]. Yan et al. reported that NBP reduces the damage of oxidative stress following CIRI in rats. Its antioxidative effect was due to inhibition of the overproduction of peroxynitrite, superoxide, and nitric oxide [28]. This effect was also proven in other disease models, such as Parkinson's disease and macrovascular diseases. In our study, higher levels of SOD were observed in mice treated with NBP compared with mice given soybean oil. Additionally, NBP treatment can also reduce increased MDA levels induced by CIRI. The measurement of neurological severity showed a similar effect of NBP.

Several limitations of the study must be mentioned. First, quantitative alteration in the neurocyte morphology should be measured. Second, the situation of apoptosis was not measured. Third, we lack immunostaining to visualize the proteins of ERK/pERK/GRASP65/pGRASP65.

## 5. Conclusions

Overall, NBP had a neuroprotective effect against CIRI, mediated by inhibiting the ERK signaling pathway and subsequently reducing GRASP65 phosphorylation. Our study investigated the underlying mechanisms of CIRI and indicated that NBP may represent a potential therapeutic drug with promising prospects for the treatment of CIRI.

## Data Availability

The data used to support the findings of this study are available from the corresponding author upon request.

## Abbreviations

NBP: DL-3-n-butylphthalide
ERK: Extracellular signal-regulated kinase
GRASPs: Golgi reassembly and stacking proteins
SOD: Superoxide dismutase
MDA: Malondialdehyde
ROS: Reactive oxygen species
TTC: Tetrazolium chloride.
